# Beverage–Drug Interaction: Effects of Green Tea Beverage Consumption on Atorvastatin Metabolism and Membrane Transporters in the Small Intestine and Liver of Rats

**DOI:** 10.3390/membranes10090233

**Published:** 2020-09-14

**Authors:** Hsien-Tsung Yao, Ya-Ru Hsu, Mei-Ling Li

**Affiliations:** Department of Nutrition, China Medical University, 91 Hsueh-shih Road, Taichung 404, Taiwan; a902782002@yahoo.com.tw (Y.-R.H.); u104059001@cmu.edu.tw (M.-L.L.)

**Keywords:** green tea, beverage, atorvastatin, membrane transporters, beverage–drug interaction, rats

## Abstract

Green tea (GT) beverages are popular worldwide and may prevent the development of many chronic diseases including cardiovascular disease and cancer. To investigate whether the consumption of a GT beverage causes drug interactions, the effects of GT beverage consumption on atorvastatin metabolism and membrane transporters were evaluated. Male rats were fed a chow diet with tap water or the GT beverage for 3 weeks. Then, the rats were given a single oral dose (10 mg/kg body weight (BW)) of atorvastatin (ATV), and blood was collected at various time points within 6 h. The results show that GT consumption increased the plasma concentrations (AUC_0–6h_) of ATV (+85%) and 2-OH ATV (+93.3%). GT also increased the 2-OH ATV (+40.9%) and 4-OH ATV (+131.6%) contents in the liver. Decreased cytochrome P450 (CYP) 3A enzyme activity, with no change in P-glycoprotein expression in the intestine, was observed in rats treated with GT. Additionally, GT increased hepatic CYP3A-mediated ATV metabolism and decreased organic anion transporting polypeptides (OATP) 2 membrane protein expression. There was no significant difference in the membrane protein expression of OATP2B1 and P-glycoprotein in the intestine and liver after the GT treatment. The results show that GT consumption may lower hepatic OATP2 and, thus, limit hepatic drug uptake and increase plasma exposure to ATV and 2-OH ATV.

## 1. Introduction

Green tea (GT) is a popular beverage in Asian countries. The bioactive phytonutrients responsible for the pharmacological activities of GT are believed to include tea polyphenols, known as catechins. The major catechins in GT include epicatechin (EC), epigallocatechin (EGC), epicatechin gallate (ECG), and epigallocatechin gallate (EGCG) [1]. Regular GT consumption may reduce the risk of cardiovascular disease and cancer, possibly due to its tea polyphenols [2].

The cytochrome P450 (CYP)3A members are the most abundant CYP enzymes in the human small intestine and liver and are particularly important in drug metabolism and elimination [3,4]. P-glycoprotein (P-gp), an efflux membrane transporter expressed on the apical membrane of epithelial cells in various tissues, including the enterocyte brush border and liver, is one of the important membrane transporters in drug elimination. P-gp limits the bioavailability of many drugs by removing the drugs from the enterocytes into the intestinal lumen and from the liver into bile [5]. Increased P-gp may prevent the accumulation of drugs or toxic compounds in tissues. In opposition to P-gp, organic anion-transporting polypeptides (OATPs) are uptake membrane transporters that are localized on the cell membranes of intestinal, liver, and other tissues. OATP2B1 and OATP2 (OATP1a1) are expressed on the basolateral membrane at high levels in the intestine [6] and the liver [7], respectively.

Recent evidence has suggested that modulations of the activities of CYP3A, P-gp, and OATPs in the intestine or liver may be involved in food/beverage–drug interactions [8,9,10]. Studies have indicated that the inhibition of CYP3A or P-gp in the intestine or liver by the consumption of grapefruit juice increases the bioavailability of several orally administered drugs, including lovastatin, simvastatin, and atorvastatin [10,11]. However, the inhibition of OATPs by grapefruit juice may increase, have no effect on, or decrease plasma drug levels in the clinic, probably dependent on the inhibition of OATPs in enterocytes or hepatocytes [12]. These results indicate that, in addition to CYP3A and P-gp, OATPs may also play a key role in interactions between foods/beverages and drugs.

The consumption of GT beverages has been reported to increase hepatic CYP1A1 and 1A2 activities and induce UDP-glucuronosyltransferase (UGT) [13,14]. However, the effect of GT or GT polyphenols on hepatic CYP3A activity is controversial. CYP3A activity was shown to be decreased [15,16], unchanged [17], or up-regulated [18,19] by GT or GT polyphenols. EGCG is likely to be one major catechin in GT that inhibits CYP3A and P-gp in the intestine and liver, resulting in a higher plasma drug concentration [20]. However, relatively less information is available about the effect of GT consumption on the function of OATPs. Unlike high doses of GT extract or tea polyphenols that may inhibit drug metabolism enzymes and membrane transporters in experimental studies, appropriate GT consumption or supplementation with modest levels of GT extract may not cause clinically significant effects on the disposition or elimination of drugs metabolized by CYP/UGT enzymes or P-gp [16,21]. Nevertheless, the disposition of drugs that are substrates of OATP membrane transporters may be influenced by GT extract consumption [22].

Atorvastatin (ATV), a 3-hydroxy-3-methylglutaryl-CoA reductase inhibitor, is one of the most potent statins for the treatment of hypercholesterolemia, especially for lowering plasma low-density lipoprotein cholesterol [23]. The absolute bioavailability of ATV in humans is approximately 14%, and the low plasma ATV concentration is attributed to intensive clearance in the intestinal mucosa and/or first-pass metabolism in the liver [24]. ATV is metabolized primarily by CYP3A in the liver to form two active metabolites, ortho-hydroxy atorvastatin (2-OH ATV) and para-hydroxy atorvastatin (4-OH ATV). These two metabolites may undergo further glucuronidation by UGT. Both ATV and its metabolites are exclusively excreted in the bile, indicating that hepatic metabolism and biliary excretion are the major routes of elimination [25]. ATV has been demonstrated to be a substrate for CYP3A, P-gp, and OATP2/2B1 [26,27,28,29].

To date, commercially available sugar-containing tea beverages have become more popular worldwide. The sugar supplemented in GT can reduce the bitterness of the taste and improve the bioavailability of some catechins by enhancing intestinal uptake [30]. To date, however, the possible GT beverage–drug interaction is unclear. Therefore, in this study, we investigated the effects of GT beverage consumption on the levels of ATV and its major active metabolite, 2-OH ATV, in rat plasma and liver. CYP3A activity and the expression of membrane transporters, P-gp, OATP2, and/or OATP2B1 in the liver and intestine were also evaluated.

## 2. Materials and Methods

### 2.1. Chemicals and Reagents

Atorvastatin (ATV) was obtained from Waterstone Technology, LLC (Carmel, IN, USA). 2-OH-ATV and 4-OH ATV were purchased from Santa Cruz Biotechnology, Inc. (Santa Cruz, CA, USA). NADPH and heparin were obtained from Sigma-Aldrich (St. Louis, MO, USA). Testosterone was obtained from Sigma-Aldrich (St. Louis, MO, USA). Midazolam, 1-OH midazolam, and 6-β-hydroxytestosterone were purchased from Ultrafine Chemicals (Manchester, UK). All other chemicals and reagents were of analytical grade and were obtained commercially.

### 2.2. Green Tea Beverage

**Figure 1:**
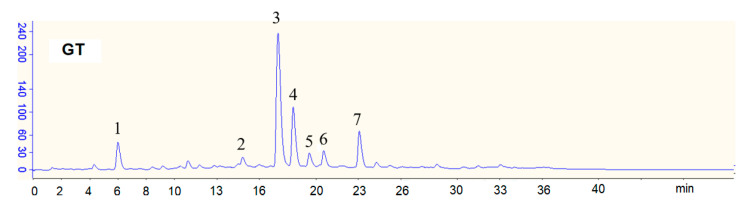
HPLC chromatograms of green tea (GT) beverage. Peaks: 1, gallic acid (GA); 2, epigallocatechin (EGC); 3, caffeine; 4, epigallocatechin gallate (EGCG); 5, epicatechin (EC); 6, gallocatechin gallate (GCG); 7, epicatechin gallate (ECG).

The green tea (GT) beverage was obtained commercially (Uni-President Enterprises Corp, Taitsung, Taiwan). The concentrations of sugars, including glucose, sucrose, and fructose, in the GT were determined using a commercial assay kit (Megazyme, Bray, Ireland). The total phenolics in the GT beverage were determined by a colorimetric method using Folin–Ciocalteu reagent, and gallic acid was used as a standard [31]. The data are expressed in μg of gallic acid equivalent/mL. The concentrations of gallic acid, caffeine, and catechins in the GT were determined by high-performance liquid chromatography (HPLC) [32] (Figure 1). Seven components in the GT could be determined and quantitated. The GCG present in the GT beverage is due to the epimerization of EGCG during autoclaving [32].

### 2.3. Animals and Treatment

To investigate the effects of GT consumption on the levels of ATV and its major metabolite, 2-OH ATV, in the plasma and liver, 4-week old male Sprague-Dawley (SD) rats, were obtained from BioLASCO, Yilan, Taiwan. After adaptation to a pelleted diet for 1 week, the rats were randomly divided into two groups with 6 animals. A standard pelleted chow diet with tap water (control group) or GT beverage (GT group) was given ad libitum to the rats. The tap water or GT beverage was the sole source of drinking fluid in each group and was replaced daily. Food and drinking fluid were available for 3 weeks. The drinking volume from each cage was measured every day. Body weight was determined every week. The rats were housed in individual plastic cages in a room with a 12 h light-and-dark cycle and kept at 23 ± 1 °C and 60 ± 5% relative humidity.

At the end of the experiment, the rats were fasted overnight and orally administered 10 mg/kg body weight (BW) of ATV in a formulation of Cremophor EL/ethanol/water (30:10:60, *v/v/v*). The volume of dosing solution administered was adjusted according to body weight. Blood samples were collected (∼150 μL) from each animal via the rat tail vein at 0 (prior to dosing), 15, and 30 min and at 1, 2, and 4 h after dosing. The animals were sacrificed at 6 h after the oral dosing of ATV. Blood samples were collected by exsanguination via the abdominal aorta while under carbon dioxide (70:30; CO_2_/O_2_) anesthesia. Plasma was separated from the blood by centrifugation (3000× *g* for 20 min at 4 °C), and heparin was used as the anticoagulant agent. The plasma alanine aminotransferase level was determined by using a commercial kit (Redox Laboratories, Antrim, UK). The rat liver was excised, weighed, and stored at −80 °C until analysis. The duodenal portion of the intestine was incubated with PBS buffer containing protease inhibitors for more than 5 min and then scraped with a glass slide to remove the mucosa [33]. The concentrations of ATV and ATV metabolites, in the plasma and liver, were determined by HPLC/mass spectrometry (MS) [34]. This study (No: 102-55-N) was approved by the Animal Center Management Committee of China Medical University, Taiwan. The animals were maintained in accordance with the guidelines for the care and use of laboratory animals as issued by the ethics committee.

### 2.4. Determination of ATV and ATV Metabolites in Plasma and Liver

ATV and 2-OH ATV in the plasma and liver were determined by HPLC/MS [34]. A 50 μL volume of plasma was mixed with 100 μL of acetonitrile. The mixture was vortexed and then centrifuged at 21,000× *g* for 20 min. The supernatant was used for HPLC/MS analysis. The blank plasma containing various concentrations of ATV (1–1000 ng/mL) or 2-OH ATV (5–1000 ng/mL) was mixed with acetonitrile to prepare the calibration curve. The calibration curves were linear, with correlation coefficients ≥0.99. 

To determine the ATV, 2-OH ATV, and 4-OH ATV contents in the liver, liver homogenate was mixed with acetonitrile (1:2; *v/v*), vortexed, and then centrifuged by the same procedure as described above. The supernatant was used for HPLC/MS determination. To prepare the samples for the calibration curve, the blank liver homogenate containing various concentrations of ATV (13–3704 ng/g), 2-OTV (14–1111 ng/g), and 4-OH ATV (14–1111 ng/g) was mixed with acetonitrile (1:2; *v/v*) and processed according to the same procedure as described above. The calibration curves were linear, with correlation coefficients ≥ 0.99. 

### 2.5. HPLC/MS Analysis

An Agilent 1100 Series HPLC/MS system (Palo Alto, CA, USA) and a Zorbax Eclipse XDB-C8 (5 μm, 150 × 3.0 mm i.d., Agilent) column were used to analyze the ATV, 2-OH, and 4-OH ATV. The mobile phase consisted of Solvent A (acetonitrile) and Solvent B (10 mM ammonia acetate +0.1% formic acid). The flow rate was 0.5 mL/min. The gradient system used to separate the ATV, 2-OH ATV, and 4-OH ATV was as follows: 70% B (0 min), 70% B to 50% B (0–1 min), 50% B to 2% B (1–3 min), 2% B (3–9 min), 2% B to 70% B (9–10 min), and 70% B (10–15 min). The total running time was 15 min. The positive selected ion monitoring (SIM) mode was used. The retention times of the ATV, 2-OH ATV, and 4-OH ATV were as follows: ATV, 5.0 min; 2-OH ATV, 9.6 min; 4-OH ATV, 9.8 min. Ions representing the positive mode ([M-H]^+^: ATV at *m/z* 181; [M-H]^+^: 2-OH and 4-OH ATV at *m/z* 272.4) were selected, and the peak areas were measured. The concentrations of ATV and ATV metabolites in the rat plasma and liver were quantitated with the calibration curves of the authentic standard.

### 2.6. CYP Enzyme Activity Assays

The liver was homogenized (1:4, *w/v*) in 0.1 M phosphate buffer (pH 7.4) containing 1 mM EDTA. The homogenate was used for two-step centrifugation to prepare liver microsomes [34]. The activities of CYP3A and UGT were determined in microsomes isolated from the liver or mucosa of the duodenum portion. The metabolite formed from each enzymatic reaction was determined by an HPLC/MS method [34]. Testosterone (60 μM) and midazolam (2.5 μM) were, respectively, used as the CYP3A probe substrates for the determination of the activities of testosterone 6β-hydroxylation and midazolam 1-hydroxylation. The microsomal proteins in the microsomal incubation were at 0.2 mg/mL. The incubation time for the metabolic reaction of testosterone 6β-hydroxylation (CYP3A) was 15 min. The corresponding value for midazolam 1-hydroxylation (CYP3A) was 5 min. 

### 2.7. Immunoblotting Analysis

Western blot analysis was performed using a previously reported method [34]. The homogenates prepared from the mucosa and liver or mucosa microsomes of each group with equal amounts of protein were separated by SDS-PAGE and then transferred to polyvinylidene difluoride membranes. After blocking, the membranes were hybridized with antibodies against anti-P-gp (Abcam, Cambridge, UK), anti-OATP2B1 (GeneTex Inc, Irvine, CA, USA), anti-OATP2 (Merck Millipore, MA, USA), and anti-CYP3A1 (Calbiochem, Darmstadt, Germany) at 4 °C with gentle agitation overnight. After washing twice with PBST, the membranes were incubated with a horseradish peroxidase (HRP)-conjugated secondary antibody (Abcam, Cambridge, UK) at room temperature for 1 h. Equal loading across the lanes was confirmed by staining the blot with Ponceau S solution. 

### 2.8. Determination of Caffeine Levels in Plasma and Liver

Plasma or liver homogenate (prepared as described above) was mixed with acetonitrile (1:2, *v/v*), vortexed, and then centrifuged at 10,000× *g* for 20 min. The supernatant was used for HPLC/MS analysis [35].

### 2.9. Determination of Fat Contents in the Liver

Lipids were extracted from the liver by the method of Folch et al. [36] and emulsified with Triton X-100 [37]. The total cholesterol and triglyceride contents in the liver were determined by using commercial kits (Audit Diagnostics, Cork, Ireland).

### 2.10. Statistical Analysis

One-way ANOVA (SAS Institute, Cary, NC, USA) was used to calculate statistical differences among groups, which were evaluated again by independent-sample t-tests. The differences were regarded as significant at *p* < 0.05. 

## 3. Results and Discussion

### 3.1. Constituents of the GT Beverage

As shown in Figure 1, seven tea components including gallic acid, caffeine, and five catechins (epigallocatechin: EGC; epigallocatechin gallate: EGCG; epicatechin: EC; gallocatechin gallate: GCG; ECG: epicatechin gallate) were determined and separated by high-performance liquid chromatography (HPLC).

Table 1 shows the concentrations of selected tea components in the GT beverage, including gallic acid (GA), catechins, caffeine, and sugars (glucose, sucrose, and fructose). EGCG is the most abundant catechin in the GT beverage. Sucrose is the most abundant sugar in the GT beverage. The caffeine concentration in the GT beverage was 252.5 μg/mL. The total phenolics content in the GT beverage was 567.0 μg of gallic acid equivalent/mL.

### 3.2. Body Weight, Tissue Weight, and Volume of Drinking Fluid

The consumption of the GT beverage for 3 weeks did not significantly affect body weight (control group: 286.6 ± 6.1 g; GT group: 295.8 ± 18.1 g) or relative kidney weight (control group: 0.8 ± 0.1 g/100 g BW; GT group: 0.8 ± 0.1 g/100 g BW) compared with those values in the control group. The rats given GT consumed significantly (*p* < 0.05) more fluid than those given tap water (control group: 22.6 ± 5.8 mL/day/rat; GT group: 40.6 ± 9.5 mL/day/rat). Besides, GT consumption mildly increased (*p* < 0.05) relative liver weights (control group: 3.0 ± 0.1 g/100 g BW; GT group: 3.3 ± 0.1 g/100 g BW). GT consumption for 3 weeks had no effect on plasma alanine aminotransferase activity, indicating that the consistent consumption of GT could not induce hepatotoxicity (*p* > 0.05).

### 3.3. Pharmacokinetics of ATV after 3 Weeks of GT Consumption in Rats

**Figure 2:**
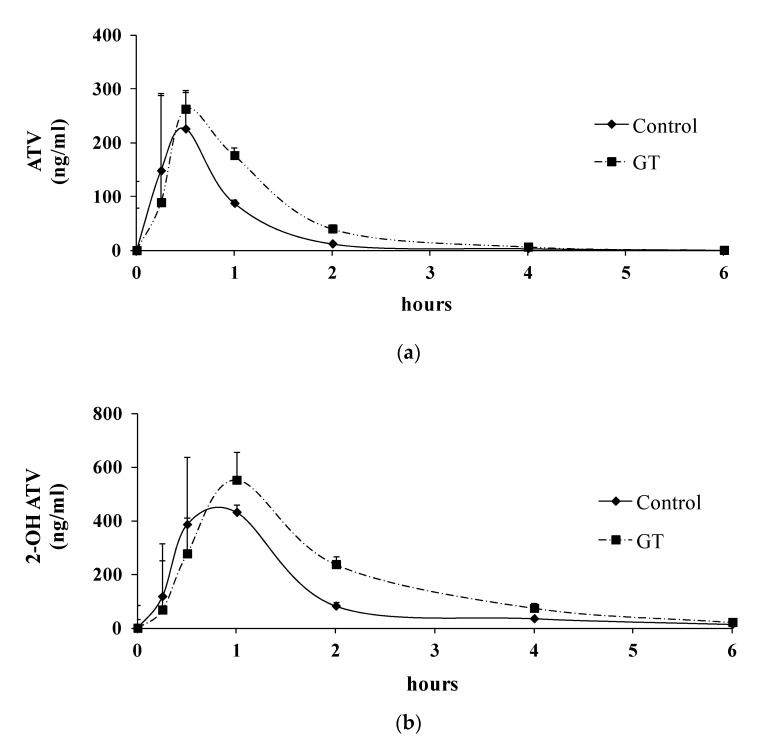
Plasma concentration–time profiles of atorvastatin (ATV) (**a**) and 2-OH ATV (**b**) after 3 weeks of GT treatment in rats. The atorvastatin was administered orally (10 mg/kg body weight) to rats after the consumption of GT for 3 weeks. Values at each time point are expressed as the mean ± SD (*n* = 6). ●: control group; ■: GT group.

After 3 weeks of GT consumption, the plasma drug concentrations of ATV and 2-OH ATV were determined, as presented in Figure 2, and the pharmacokinetic parameters (AUC_(0–6 h)_, T_max_, and C_max_) of ATV and 2-OH ATV are presented in Table 2. The results show that GT consumption increased the plasma concentrations (AUC_(0–6 h)_) of ATV and 2-OH ATV.

The results show an increase in the AUC_(0–6 h)_ values of ATV (+85.1%) and 2-OH ATV (+93.3%) after 3 weeks of GT consumption. The C_max_ and T_max_ values are the important parameters for the absorption phase of oral drugs [38]. Although an increase in the C_max_ values for ATV (C_max_: +30.5%) and 2-OH ATV (C_max_: +19.6%) was observed in the GT group, the increase in the C_max_ values for both ATV and 2-OH ATV with GT did not represent a statistically significant difference (*p* > 0.05). The T_max_ values for ATV and 2-OH ATV were comparable between the control group and GT group (*p* > 0.05).

### 3.4. Effect of GT Consumption on Hepatic Contents of ATV, 2-OH ATV, and 4-OH ATV

**Figure 3:**
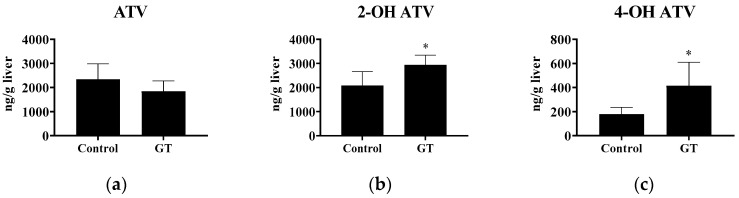
Effect of GT consumption on the hepatic contents of ATV, 2-OH ATV, and 4-OH ATV. The analytical methods are described in Materials and Methods. The values are given as the mean ± SD (*n* = 6). * Significantly different from the control group, *p* < 0.05.

After 3 weeks of GT consumption, ATV (10 mg/kg BW) was administrated orally to each rat. The ATV, 2-OH ATV, and 4-OH ATV levels in the liver were determined. As shown in Figure 3, the results show that GT had no effect (*p* > 0.05) on the hepatic ATV level after 6 h of ATV administration. However, GT increased the 2-OH ATV (+40.9%) and 4-OH ATV (+131.6%) contents in the liver (*p* < 0.05).

### 3.5. Effect of GT Comsumption on the Activities of CYP3A and UGT and Protein Expression of Drug Transporters in the Small Intestine

**Figure 4:**
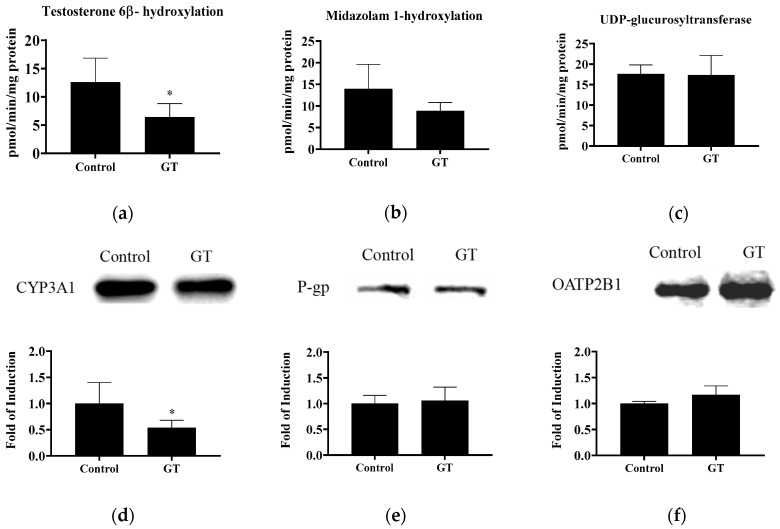
The activities of testosterone 6β-hydroxylation (CYP3A) (**a**), midazolam 1-hydroxylation (CYP3A) (**b**), and UDP-glucuronosyltransferase (**c**) and protein expression of CYP3A1 (**d**), P-gp (**e**), and OATP2B1 (**f**) in the intestine. The analytical methods are described in Materials and Methods. The values are given as the mean ± SD (*n* = 6). * Significantly different from the control group, *p* < 0.05.

The activities of CYP3A and UGT in the small intestine are shown in Figure 4. In this study, GT lowered intestinal CYP3A activity, as indicated by the reduction of testosterone 6β-hydroxylation (*p* < 0.05) and midazolam 1-hydroxylation (*p* < 0.1). CYP3A1 protein expression in the intestine was also reduced by GT (*p* < 0.05). Intestinal UGT activity was not significantly changed by GT (*p* > 0.05). No significant difference (*p* > 0.05) was noted in the protein expression of intestinal P-gp and OATP2B1 between the control group and GT group. 

Recently, several studies have demonstrated that the activities of CYP3A, P-gp, and selected OATPs in the intestine and/or liver may be involved in food/beverage–drug interactions [8,9,10]. In the intestine and liver, GT extract or EGCG may inhibit both CYP3A activity and P-gp, resulting in increasing intestinal drug absorption and plasma drug concentrations [16,20]. The inhibition of intestinal OATP2B1 may reduce drug absorption (e.g., rosuvastatin) and lower plasma drug concentrations [21]. In the liver, reduced OATP2 activity and protein expression may lower hepatic atorvastatin uptake and increase plasma drug exposure [28]. Atorvastatin is a substrate of CYP3A, P-gp, and OATP2/2B1 [26,27,28,29]. Additionally, atorvastatin and its metabolites are eliminated primarily in bile following hepatic metabolism [25]. In the present study, the 3-week consumption of the GT beverage resulted in no change in the protein expression of P-gp and OATP2B1 in the intestine, suggesting that the efflux or uptake of atorvastatin in the intestinal mucosa may have been unchanged. Besides, GT consumption induced decreased intestinal CYP3A activity in rats. This result suggests that the intestinal CYP3A-mediated atorvastatin metabolism was reduced, thus the lowered limit of atorvastatin elimination. Therefore, GT may increase the intestinal absorption of atorvastatin. This speculation could be further supported by a mildly higher C_max_ value, the parameter for the absorption phase of oral drugs [38], after the GT treatment (Table 2). 

### 3.6. Effect of GT Comsumption on the Activities of CYP3A and UGT and Protein Expression of Drug Transporters in the Liver

**Figure 5:**
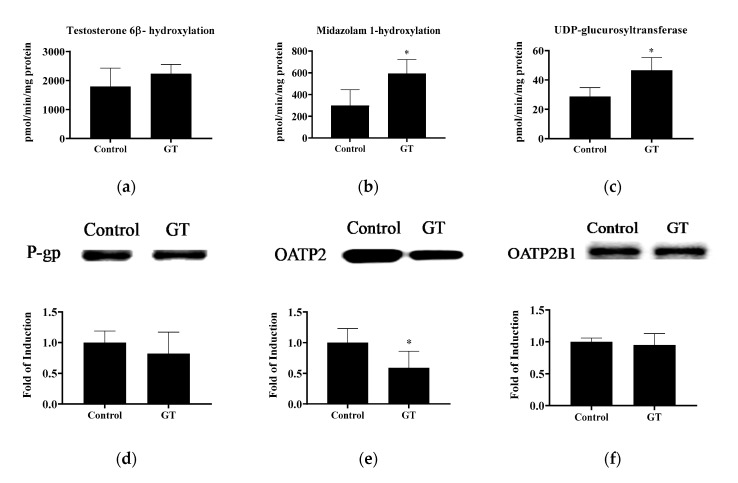
The activities of testosterone 6β-hydroxylation (CYP3A) (**a**), midazolam 1-hydroxylation (CYP3A) (**b**), and UDP-glucuronosyltransferase (**c**) and protein expression of P-gp (**d**), OATP2 (**e**) and OATP2B1 (**f**) in the liver. The analytical methods are described in Materials and Methods. The values are given as the mean ± SD (*n* = 6). * Significantly different from the control group, *p* < 0.05.

The activities of CYP3A and UGT and drug transporters in the liver are shown in Figure 5. GT treatment increased the hepatic activities of midazolam 1-hydroxylation (*p <* 0.05), testosterone 6*β*-hydroxylation (*p <* 0.1), and UGT (*p <* 0.05). These results indicate that GT consumption may enhance ATV metabolism in the liver. Notably, GT treatment lowered (*p <* 0.05) OATP2 protein expression in the liver (control group: 1.01 ± 0.23; GT group: 0.59 ± 0.27). No significant difference (*p* > 0.05) was noted in the protein expression of hepatic P-gp and OATP2B1 between the control group and GT group.

Nishikawa et al. [19] reported that the consumption of GT extract has opposing effects on CYP3A activity in the small intestine and the liver. In their study, GT extract reduced intestinal CYP3A activity, while hepatic CYP3A activity was up-regulated. The increased hepatic CYP3A activity after GT or GT extract treatment may be related to the different experimental conditions, including the different species of tea used and the experimental periods of time [18,19]. In this study, the increased hepatic activities of CYP3A and UGT after GT treatment suggested that atorvastatin was intensively metabolized in the liver. However, GT had no effect on hepatic P-gp protein expression. Therefore, the increased hepatic CYP3A-catalyzed 2-OH ATV and 4-OH ATV formation with no change in the P-gp efflux protein level may result in the accumulation of both ATV active metabolites in the liver (Figure 3). Besides, lower hepatic OATP2 (−45.8%) after GT consumption may limit the uptake of ATV and 2-OH ATV by hepatocytes from the circulation and, thus, increase their plasma exposure. Taken together, it is suggested that the inhibition of hepatic OATP2-mediated drug uptake by GT is the major determinant of the increased plasma concentrations (AUC_0-6h_) of ATV and 2-OH ATV. 

Recent studies have indicated that OATP2B1 mediates the hepatic and intestinal uptake of many drugs and endogenous compounds, resulting in food–drug interactions [39]. Green tea catechins, especially ECG and EGCG, are both substrates and inhibitors of several OATPs, including OATP2B1 in Chinese hamster ovary (CHO) cells [40]. However, a 5-fold stimulation of OATP1B3 estrone-3-sulfate uptake by 100 μM EGCG was also observed in the same experimental model. These results indicate that EGCG may exert differential effects, including inhibition and induction, on selected OATPs. In this study, there were no significant differences in OATP2B1 protein expression in the intestine or liver between the control and GT groups. It is possible that multiple components coexisting in GT may compensate for the inhibitory effect of EGCG on OATP2B1 in the intestine. 

### 3.7. Effect of GT on Hepatic Contents of Triglycerides and Cholesterol

In this study, the daily consumption of the GT beverage instead of drinking water mildly increased (*p* < 0.05) the hepatic triglyceride level (control group: 9.8 ± 0.4 mg/g; GT group: 12.4 ± 2.5 mg/g) without affecting the cholesterol level (control group: 3.9 ± 0.4 mg/g; GT group: 3.8 ± 0.4 mg/g). After the 6 h administration of a single dose (10 mg/kg BW) of atorvastatin, plasma cholesterol was not changed between the two groups (control group: 72 ± 8 mg/dL; GT group: 68 ± 10 mg/dL). These results suggest that the increased ATV and 2-OH ATV levels in the plasma or ATV metabolites in the liver after 6 h of ATV administration might have no effect on cholesterol levels in the plasma and liver of normal rats. A mild increase in the hepatic triglyceride level was probably due to the sugars in the GT beverage, which may increase triglyceride synthesis [41]. Therefore, the sugars supplemented in the GT beverage may counteract the lipid-lowering effect of GT or GT polyphenols. The caffeine level in the plasma after GT beverage consumption was maintained at 3.9 ± 3.4 μg/mL. The corresponding value in the liver was 234.8 ± 147.0 μg/g.

The increased fluid intake (approximately 1.8-fold) of the rats in the GT group might have been due to the sweetness of the beverage compared with the water consumed by the control group. In this study, a mildly increased (+26.5%) hepatic triglyceride content was observed in the GT group. Our data suggested that the long-term consumption of sugar-containing GT beverages (5.6% sugar; *w/v*) may increase triglyceride synthesis in the liver [41]. It has been reported that long-term (90 days) feeding with a high-sucrose diet (60% instead of starch in the diet, w/w) reduced the hepatic activities of CYP1A1, CYP3A2, and glutathione S-transferase [42]. Therefore, in this study, the effect of sugar in GT on ATV metabolism and disposition may not be excluded. To our knowledge, less information is known regarding the effect of sugar intake in drinking water or beverages on drug-metabolizing enzymes and membrane transporters. 

## 4. Conclusions

The consistent consumption of a GT beverage may increase the plasma concentrations of ATV and 2-OH ATV and enhance the hepatic levels of 2-OH and 4-OH ATV. GT differentially affects CYP3A activity in the small intestine and liver. The daily drinking volume of the GT beverage in this study was approximately 1328 mL in human terms (60 kg BW), which may be achieved by consuming 5.5 cups of GT. Since CYP3A4 in humans is responsible for the metabolism of more than 50% of clinical drugs, GT beverage–atorvastatin interactions may be considered clinically relevant in these populations. Notably, GT beverage consumption had no effects on the protein expression of membrane transporters, P-gp, and OATP2B1 in the small intestine and liver. However, GT lowered hepatic OATP2 protein expression, may limit hepatic drug uptake, and, thus, may increase the plasma drug exposure of ATV and 2-OH ATV. Since the GT beverage contains sugars and GT, the effects of individual constituents in the GT beverage—such as sugars or GT—on ATV metabolism, pharmacokinetics, and membrane transporters will be further investigated in future studies.

## Figures and Tables

**Table 1:** Constituents in green tea ^1^.

	Gallic Acid (GA)	Epigallocatechin (EGC)	Epigallocatechin Gallate (EGCG)	Epicatechin (EC)	Gallocatechin Gallate (GCG)	Epicatechin Gallate (ECG)	Total Catechins ^2^
Polyphenols (μg/mL)	3.8	282.6	208.8	62.9	45.6	67.0	670.7
Sugars (g/L)	**Glucose** 7.1	**Sucrose** 38.9	**Fructose** 9.6	**Total sugars ^3^** 55.6			

^1^ Values were expressed as the means of duplicate data. ^2^ Total catechins: EGC + EGCG + EC + GCG + ECG. ^3^ Total sugars: Glucose + Sucrose + Fructose.

**Table 2:** Pharmacokinetic parameters of ATV and 2-OH ATV (oral administration) after the consumption of the GT beverage for 3 weeks in rats.

	AUC (0-t) (ng/mLxh)	C_max_ (ng/mL)	T_max_ (min)
ATV			
Control group	176.5 ± 101.1	235.5 ± 136.1	27.0 ± 6.7
GT group	326.8 ± 88.7 *	307.4 ± 157.7	33.0 ± 16.4
2-OH ATV			
Control group	548.3 ± 134.2	466.2 ± 225.7	48.0 ± 16.4
GT group	1059.8 ± 185.0 *	557.5 ± 125.7	72.0 ± 26.8

AUC: area under the plasma drug concentration curve. t = 6 h. C_max_: value of the maximal observed concentration. T_max_: the time at which the maximum concentration is observed. * Significantly different from the control group, *p* < 0.05.
